# Crizotinib Shows Antibacterial Activity against Gram-Positive Bacteria by Reducing ATP Production and Targeting the CTP Synthase PyrG

**DOI:** 10.1128/spectrum.00884-22

**Published:** 2022-06-08

**Authors:** Yun-Dan Zheng, Tairan Zhong, Haiming Wu, Nan Li, Zuye Fang, Linlin Cao, Xing-Feng Yin, Qing-Yu He, Ruiguang Ge, Xuesong Sun

**Affiliations:** a MOE Key Laboratory of Tumor Molecular Biology and Key Laboratory of Functional Protein Research of Guangdong Higher Education Institutes, Institute of Life and Health Engineering, College of Life Science and Technology, Jinan University, Guangzhou, China; b State Key Laboratory of Biocontrol, College of Life Sciences, Sun Yat-Sen University, Guangzhou, China; UNMC

**Keywords:** quantitative proteomics, crizotinib, Gram-positive bacteria, drug repurposing

## Abstract

Infections caused by drug-resistant bacteria are a serious threat to public health worldwide, and the discovery of novel antibacterial compounds is urgently needed. Here, we screened an FDA-approved small-molecule library and found that crizotinib possesses good antimicrobial efficacy against Gram-positive bacteria. Crizotinib was found to increase the survival rate of mice infected with bacteria and decrease pulmonary inflammation activity in an animal model. Furthermore, it showed synergy with clindamycin and gentamicin. Importantly, the Gram-positive bacteria showed a low tendency to develop resistance to crizotinib. Mechanistically, quantitative proteomics and biochemical validation experiments indicated that crizotinib exerted its antibacterial effects by reducing ATP production and pyrimidine metabolism. A drug affinity responsive target stability study suggested crizotinib targets the CTP synthase PyrG, which subsequently disturbs pyrimidine metabolism and eventually reduces DNA synthesis. Subsequent molecular dynamics analysis showed that crizotinib binding occurs in close proximity to the ATP binding pocket of PyrG and causes loss of function of this CTP synthase. Crizotinib is a promising antimicrobial agent and provides a novel choice for the development of treatment for Gram-positive infections.

**IMPORTANCE** Infections caused by drug-resistant bacteria are a serious problem worldwide. Therefore, there is an urgent need to find novel drugs with good antibacterial activity against multidrug-resistant bacteria. In this study, we found that a repurposed drug, crizotinib, exhibits excellent antibacterial activity against drug-resistant bacteria both *in vivo* and *in vitro* via suppressing ATP production and pyrimidine metabolism. Crizotinib was found to disturb pyrimidine metabolism by targeting the CTP synthase PyrG, thus reducing DNA synthesis. This unique mechanism of action may explain the decreased development of resistance by Staphylococcus aureus to crizotinib. This study provides a potential option for the treatment of drug-resistant bacterial infections in the future.

## INTRODUCTION

The dramatic increase in drug-resistant pathogens, in particular the emergence of superbugs, has seriously threatened public health (1, 2). Gram-positive drug-resistant pathogens remain among the top causes of health care-associated infections, and therefore are a high priority in drug development research (3, 4). Especially, methicillin-resistant Staphylococcus aureus is one of the most common opportunistic human bacterial pathogens and causes serious infections, including suppurative infections, pneumonia, and sepsis (5–8). The development of novel and efficient strategies to treat infectious diseases caused by Gram-positive pathogens has become an urgent task.

Compared with traditional development approaches, drug repurposing provides a faster and more effective approach to identify new antimicrobial agents (9, 10). Most repurposed drugs already have detailed information about safety and pharmacokinetic profiles available, which reduces the research cost and shortens the development period, thereby accelerating their application in clinical treatment (11). Some compounds approved by the U.S. Food and Drug Administration (FDA), such as the antifungal naftifine, antispasmodic otilonium bromide, and antirheumatic auranofin, have been reported as inhibitors of Gram-positive bacterial infections (12–15). However, these drugs are not reported to have broad-spectrum antimicrobial or antibacterial effects on clinically isolated multidrug-resistant strains. Thus, there are currently very few repurposed drugs that can be used to inhibit clinical bacterial strains.

To find more efficient antibacterial compounds, we screened FDA-approved drugs and found that crizotinib exhibited antibacterial effects against Gram-positive bacteria. Crizotinib is a novel orally active multitargeted tyrosine kinase inhibitor that inhibits anaplastic lymphoma kinase, hepatocyte growth factor receptor, and the receptor “recepteur d'origine nantais.” It is a reversible competitive inhibitor against ATP binding to the intracellular catalytic domain of these oncogenic tyrosine kinases (16, 17). However, the detailed antibacterial properties and antibacterial mechanisms of crizotinib are unknown.

Here, we found that the repurposed drug crizotinib showed good antibacterial activity against bacteria, including Gram-positive bacteria, which cannot easily develop drug resistance to it. Furthermore, it showed antibacterial synergy with clindamycin (Cli) and gentamicin (Gent). In an animal model, crizotinib increased the survival rate and reduced pulmonary inflammation in mice. Subsequently, data-independent acquisition (DIA) quantitative proteomics coupled with drug affinity responsive target stability (DARTS) showed that crizotinib exerted its antibacterial effects by targeting PyrG, thereby interfering with pyrimidine metabolism and eventually disrupting DNA synthesis. Therefore, our study suggests a promising antibacterial drug with a detailed mechanism of action that may contribute to the treatment of Gram-positive bacterial infections in the future.

## RESULTS

### Crizotinib showed good antimicrobial activity and avoided bacterial resistance.

**FIG 1:**
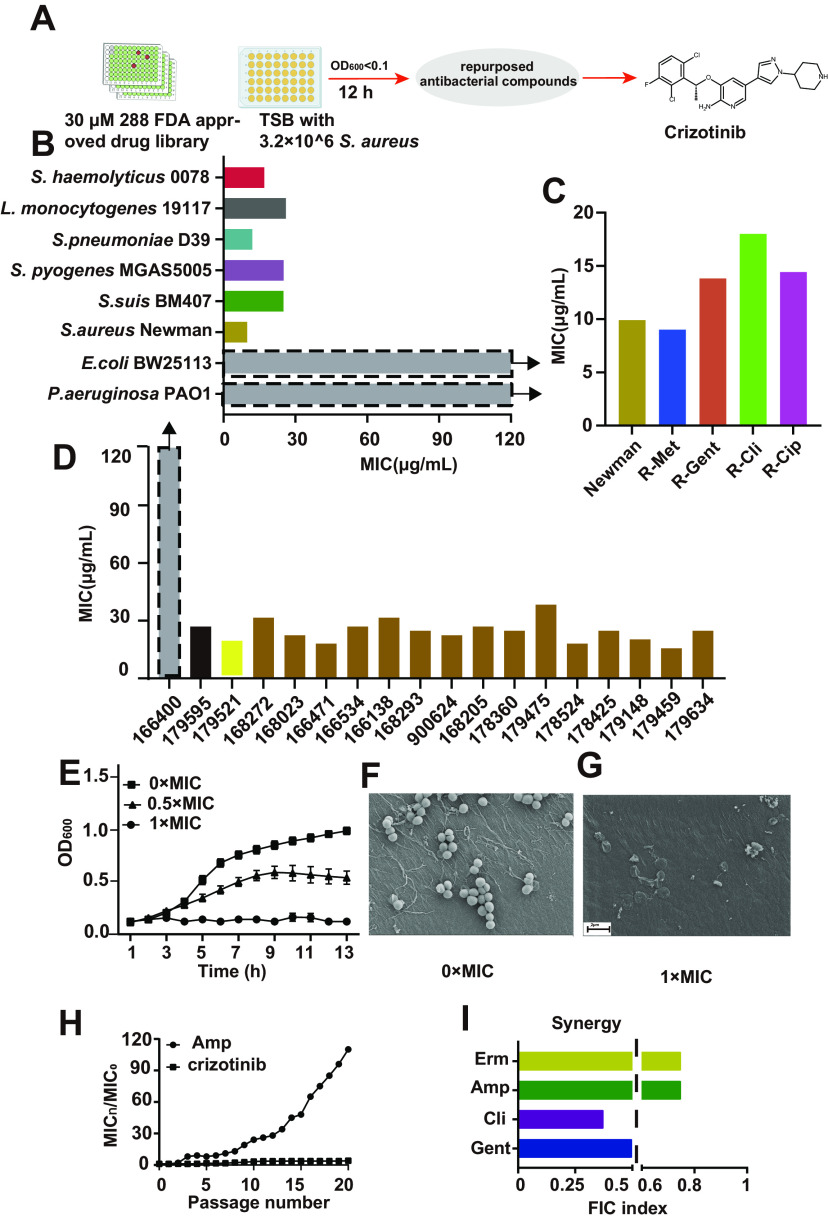
Screening of an FDA-approved small-molecule library led to the identification of crizotinib as a prospective antibacterial agent. (A) Flow chart of screening antibacterial compound in the FDA-approved drug library; (B) MIC of crizotinib against Gram-positive and Gram-negative bacteria. The MICs of E. coli BW25113 and P. aeruginosa PA101 were larger than the maximal drug concentration (120 μg/mL) tested. (C) MIC of crizotinib against the monoresistant strains; (D) MIC of crizotinib against clinically isolated multiple-resistant strains. Brown represents clinically isolated multiple-resistant S. aureus, black represents clinically isolated multiple-resistant E. faecalis, yellow represents clinically isolated multiple-resistant *S. haemolyticus* 0078, and gray represents clinically isolated multiple-resistant E. coli. The MIC of clinically isolated multiple-resistant E. coli was larger than the maximal drug concentration (120 μg/mL) tested. (E) Growth curve of S. aureus cultured in TSB medium with 0× the MIC, 0.5× the MIC, and 1× the MIC of crizotinib; (F, G) SEM images of S. aureus exposed to DMSO (control) or 1× the MIC of crizotinib; (H) development of resistance of S. aureus Newman to crizotinib and Amp; (I) synergistic antibacterial efficacy of crizotinib combined with either Gent or Cli for S. aureus.

To find novel antibacterial agents, a drug library of 288 FDA-approved medications was tested for antibacterial activity. S. aureus Newman was treated with the 288 compounds individually at an identical concentration of 30 μM for 12 h, and the inhibitory effects on bacteria were evaluated by determining the optical density at 600 nm (OD_600_). As shown in Fig. 1A, crizotinib, a novel orally active multitargeted tyrosine kinase inhibitor, was one of the most potent compounds in suppressing S. aureus Newman.

To assess the antibacterial spectrum of crizotinib, we measured the MIC of crizotinib against several pathogens commonly found in clinical settings. We found that crizotinib significantly inhibited the growth of multiple Gram-positive pathogens, including S. aureus, Streptococcus suis, Streptococcus pneumoniae, Enterococcus faecalis, Staphylococcus haemolyticus, Listeria monocytogenes, and Streptococcus pyogenes, with MICs of 9 to 26 μg/mL, but had little effect on Gram-negative bacteria (Fig. 1B). Moreover, crizotinib also hindered the growth of various single-antibiotic-resistant S. aureus strains, including ciprofloxacin-resistant (Cip^r^) strain ATCC 29213, methicillin-resistant (Met^r^) strain ATCC 29213 (MRSA-29213), clindamycin-resistant (Cli^r^) strain ATCC 29213, and gentamicin-resistant (Gent^r^) S. aureus strains, with MICs of 9 to 18 μg/mL (Fig. 1C). More importantly, it efficiently suppressed several clinically isolated multidrug-resistant strains of E. faecalis, S. aureus, and *S. haemolyticus*, with MICs of 15 to 38 μg/mL (Fig. 1D). These results suggest that crizotinib has a broad spectrum of antibacterial activity against Gram-positive pathogens, even for clinically isolated multidrug-resistant bacteria.

Furthermore, we used a representative Gram-positive bacterium, S. aureus Newman, as a model to investigate the antibacterial activity and mechanism of action for crizotinib. The growth curves of S. aureus Newman showed that the bacterial growth rate decreased in a dose-dependent manner with crizotinib (Fig. 1E). Based on the principle that more proteins are detected in bacteria, 0.5× the MIC of crizotinib was chosen for the subsequent proteomics assay in this study.

Moreover, scanning electron microscopy (SEM) was used to observe changes in bacterial morphology after crizotinib treatment. The SEM images showed that the cells in the control group had an intact structure and a smooth surface (Fig. 1F). In contrast, the cell structure of S. aureus treated with crizotinib was significantly disrupted (Fig. 1G). The results showed that crizotinib treatment damaged the cell structure of S. aureus.

The drug resistance of bacteria is also an important index to judge whether an antibacterial drug has potential for clinical application. Therefore, we tested the resistance development of S. aureus against crizotinib. S. aureus Newman was cultured in the presence of sub-MIC values of crizotinib and ampicillin (Amp [positive control]). The fold change in the MIC showed that bacteria had a low tendency for development of drug resistance to crizotinib from the first passage to the 20th passage (Fig. 1H). Contrastingly, the bacteria exhibited a 120-fold increase in the Amp MIC after approximately 20 passages. Therefore, compared with commonly used antibiotics, crizotinib has a greater advantage in avoiding bacterial drug resistance.

Using combinational therapy for antibiotics is an important method for the clinical treatment of mixed bacterial infections, multidrug-resistant bacterial infections, and severe infections (18). To achieve better antibacterial efficacy, the commonly used antibiotics such as Gent, Amp, erythromycin (Erm), and Cli were combined with crizotinib to test the antibacterial effect. The results showed that the fractional inhibitory concentration (FIC) values of crizotinib combined with antibiotics, including Cli and Gent, were ≤0.5 for S. aureus Newman. These results illustrated that crizotinib with Cli or Gent had a synergistic antibacterial effect (Fig. 1I).

### Crizotinib has a protective antibacterial effect *in vivo*.

**FIG 2:**
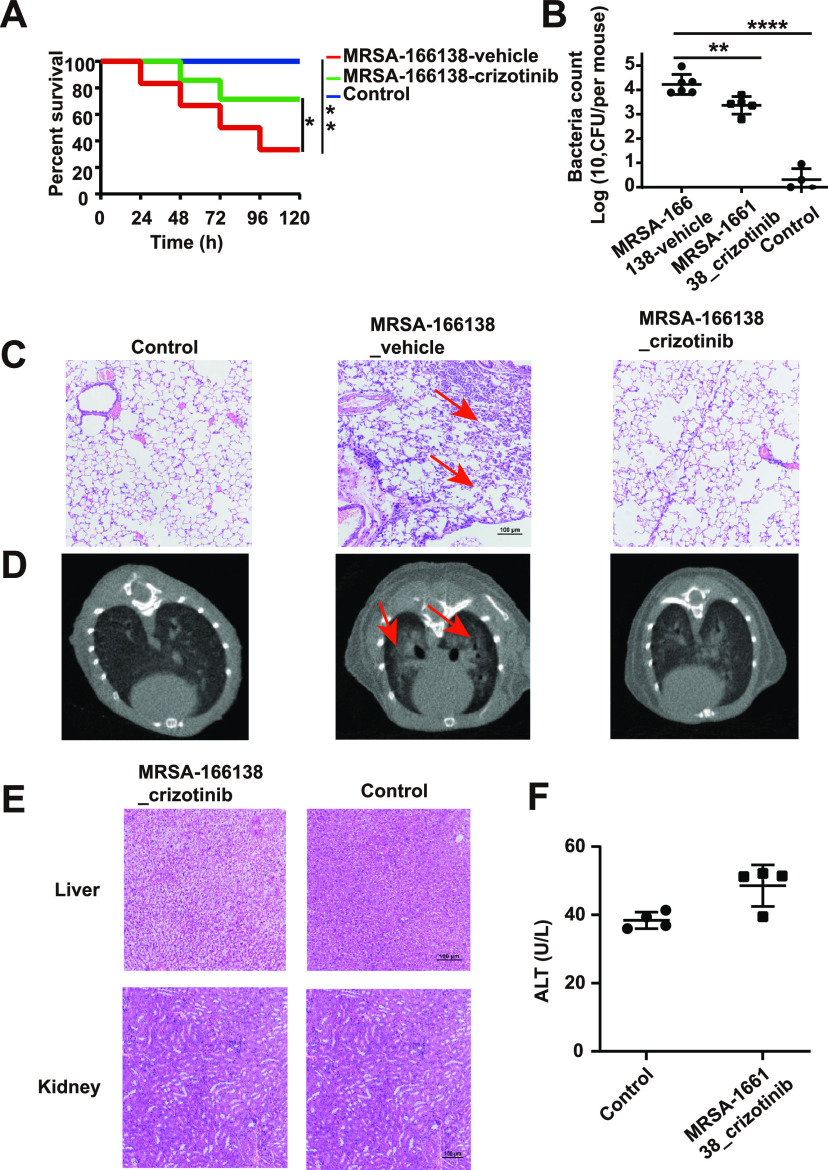
Therapeutic efficacy of crizotinib for the mice with MRSA-166138-induced pneumonia. (A) Survival rate of mice (*n* = 6); (B) CFU counts in the lung tissues at 48 h postinfection; (C) hematoxylin and eosin (HE) staining of lung collected from control group and crizotinib- and vehicle-treated groups. The arrow indicates alveoli with inflammatory cells. Scale bar, 100 μm; (D) pulmonary inflammation recorded through CT scanning. The arrow indicates pulmonary shadow; (E) HE staining of liver and kidney collected from crizotinib- and vehicle-treated mice; (F) ALT level in serum of the crizotonib- and vehicle-treated mice. *, *P* < 0.05; **, *P* < 0.01; ****, *P* < 0.0001.

To further evaluate the antibacterial effect of crizotinib *in vivo*, a mouse model of S. aureus MRSA-166138 was established. In the analysis of mouse survival rates, the median lethal dose of the MRSA-166138 strain was 1 × 10^10^ CFU in a mouse pneumonia model. After 5 days of observation, crizotinib treatment significantly improved the survival rate of mice infected with MRSA-166138 (Fig. 2A). This survival analysis illustrated that the crizotinib-treated group had a significant survival advantage over the vehicle-treated control group (mice infected with 0.5% dimethyl sulfoxide [DMSO] and phosphate-buffered saline [PBS]).

The subsequent assay evaluating bacterial load showed results consistent with the survival rate analysis. As shown in Fig. 2B, in the vehicle-treated group, where the vehicle contained DMSO (<0.5% [vol/vol]), the bacterial load in the lungs of the mice was approximately 4 × 10^4^ CFU/mouse. However, after 48 h of crizotinib treatment, the bacterial load was reduced to only 1.3 × 10^3^ CFU/mouse, showing an obvious inhibitory effect on the invasion of lung tissue by the MRSA-166138 strain. Furthermore, histopathological examination revealed that the lung tissue of the vehicle-treated group was markedly hyperemic, with a large number of inflammatory cells accumulated in the alveoli. After crizotinib treatment, the number of inflammatory cells was significantly decreased, and the structure of alveoli became relatively complete, suggesting that pulmonary inflammation was alleviated (Fig. 2C). Consistent with these findings, computed tomography (CT) image analysis revealed a significantly smaller shadow in the lungs of the crizotinib-treated group compared to the vehicle-treated group (Fig. 2D).

Moreover, to further characterize the potential of crizotinib as an antibacterial agent, the safety of crizotinib was also assessed by histopathological examination of the liver and kidney. Treatment with crizotinib did not change the cellular morphology of either the liver or kidney (Fig. 2E). Furthermore, no obvious difference in serum alanine aminotransferase (ALT) was observed between the crizotinib treatment and control groups, indicating that crizotinib has low toxicity in mice (Fig. 2F). Taken together, these results indicate that crizotinib has potential as an antibacterial agent with strong antibacterial efficacy and reliable *in vivo* safety.

### DIA-based quantitative proteomics analysis of the antibacterial mechanism of crizotinib.

**FIG 3:**
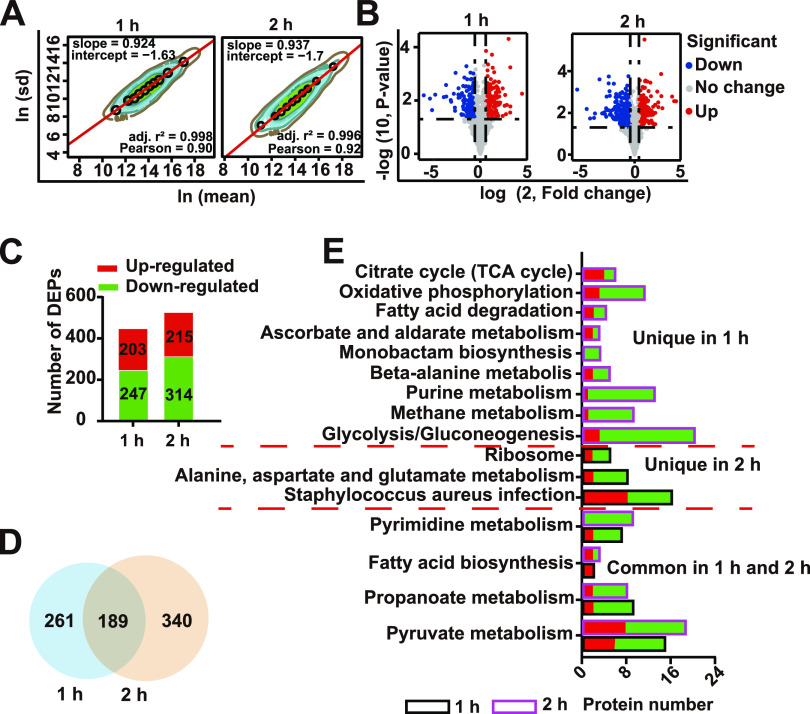
Statistical analysis of proteomic changes in S. aureus Newman induced by crizotinib. (A) PLGEM fitting based on the abundance of crizotinib-regulated proteins; (B) volcano plots of total protein in the 1-h group and 2-h group; (C) numbers of upregulated and downregulated DEPs in the 1-h group and 2-h group; (D) diagram of numbers of DEPs in the 1-h group and 2-h group; (E) KEGG enrichment analysis of DEPs.

To investigate the potential antibacterial mechanism of crizotinib against S. aureus, DIA-based quantitative proteomics was used to identify differentially expressed proteins (DEPs) between the control and groups treated with 0.5× the MIC of crizotinib for 1 h and 2 h. The power law global error model (PLGEM) algorithm was used to assess the proteomic data for the DEPs. The protein abundance of the 1-h group and 2-h group was analyzed with Pearson’s correlation coefficients of 0.90 and 0.92 and adjusted *R*^2^ values of 0.998 and 0.996, respectively (Fig. 3A), validating of the reliability of our quantitative proteomics data.

In total, 1,511 and 1,354 proteins were quantified in the 1-h and 2-h groups, respectively. As shown in volcano plots, the change tendency of the DEPs had similar distributions in the 1-h and 2-h groups (Fig. 3B), and 450 and 529 DEPs were identified in the 1-h and 2-h groups, respectively. A total of 189 DEPs were shared between the two groups. In the 1-h group, 203 upregulated and 247 downregulated proteins were identified, while 215 upregulated and 314 downregulated proteins were found in the 2-h treatment group (Fig. 3C and D). To analyze the mechanism of action of crizotinib, DEPs were subjected to further bioinformatics analysis.

Kyoto Encyclopedia of Genes and Genomes (KEGG) pathway analysis of these DEPs showed that some pathways were continuously downregulated with crizotinib treatment, including fatty acid biosynthesis and pyrimidine, propanoate, and pyruvate metabolism. In the 1-h treatment group, 0.5× the MIC of crizotinib caused downregulation of glycolysis, the purine, methane, alanine, aspartate, and glutamate metabolism pathways, and oxidative phosphorylation. Pathways involved in bacterial survival, such as ribosome, alanine, aspartate, and glutamate metabolisms, as well as S. aureus infectivity, were significantly downregulated in the 2-h treatment group (Fig. 3E).

**FIG 4:**
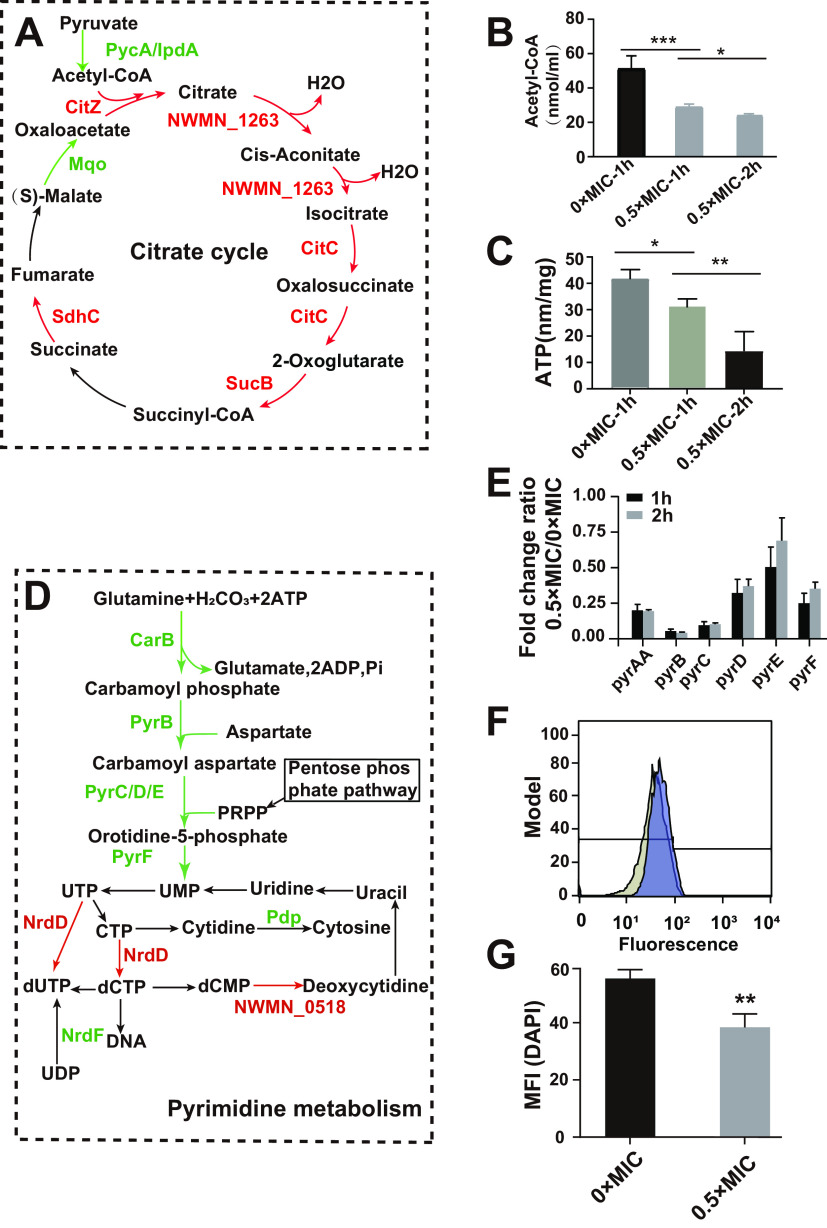
Crizotinib interferes with pyrimidine metabolism to decrease DNA synthesis. (A) Diagram showing the DEPs in citrate cycle; (B, C) acetyl-CoA and ATP levels of control S. aureus and S. aureus treated with 0.5× the MIC of crizotinib; (D) diagram showing the DEPs in pyrimidine metabolism; (E) RT-qPCR analysis of selected genes in S. aureus with crizotinib treatments for 1 h and 2 h versus that with the corresponding control group; (F) fluorescence histogram of DAPI in S. aureus with (deep purple) or without (light brown) treatment with 0.5× the MIC of crizotinib; (G) MFI of DAPI in S. aureus with or without treatment with 0.5× the MIC of crizotinib. *, *P* < 0.05; **, *P* < 0.01.

Interestingly, the DIA-based proteomics data showed that the upregulation of five proteins (NWMN_1263, CitC, SucB, SdhC, and CitZ) involved in the tricarboxylic acid (TCA) cycle and downregulation of two key proteins (PycA and IpdA) responsible for the conversion of pyruvate to acetyl coenzyme A (acetyl-CoA) occurred simultaneously after crizotinib treatment (Table 1 and Fig. 4A). Then, the quantification result showed that the amount of intracellular acetyl-CoA significantly decreased in crizotinib-treated bacteria (Fig. 4B). Meanwhile, NWMN_1263, CitC, SucB, SdhC, and CitZ, involved in the TCA cycle, were simultaneously increased in crizotinib-treated bacteria, which may need more ATP to resist the stimulation of drug. However, the ATP level in these bacteria was also decreased (Fig. 4C). Therefore, we speculated that crizotinib treatment interfered with bacterial growth by decreasing substrate material (acetyl-CoA) production and resulting in low ATP production of the TCA cycle, even though the key enzymes in TCA cycle were increased.

**TABLE 1:** DEPs involved in the citrate cycle and pyrimidine metabolism

KEGG	Protein	Crizotinib treated vs control			
		1 h		2 h	
		Fold change	*P* value	Fold change	*P* value
Citrate cycle	PycA	0.38	0.000	0.17	0.002
	lpdA	0.63	0.005	NS^*a*^	
	CitZ	5.34	0.000	NS	
	Mqo	NS		0.49	0.002
	SdhC	22.74	0.000	NS	
	SucB	NS		2.30	0.000
	CitC	2.68	0.000	3.50	0.000
	NWMN_1263	1.86	0.004	2.76	0.001

Pyrimidine metabolism	CarB	NS		0.35	0.000
	PyrB	0.67	0.006	0.28	0.000
	PyrC	0.64	0.011	0.36	0.000
	PyrD	0.40	0.002	0.00	0.002
	PyrE	2.28	0.003	0.44	0.027
	PyrF	NS		0.31	0.001
	Pdp	0.56	0.000	0.26	0.005
	NrdD	1.78	0.001	NS	
	NrdF	NS		0.43	0.015

a NS, no statistically significant difference.

More importantly, the proteomics data showed that proteins PyrC, PyrD, PyrF, PyrE, PyrB, etc., involved in pyrimidine metabolism, were simultaneously downregulated after crizotinib treatment, which may lead to decline of pentose phosphate and glutamine conversion to DNA (Table 1 and Fig. 4D). The real-time quantitative PCR (RT-qPCR) results confirmed that the mRNA expression levels of the genes encoding the above-mentioned proteins were also downregulated to different extents (Fig. 4E). We further measured the DNA content of S. aureus Newman by flow cytometry using the fluorescent dye 2-(4-amidinophenyl)-6-indolecarbamidine dihydrochloride (DAPI). The calculated mean fluorescence intensity (MFI) showed a decrease in DNA content in S. aureus upon 0.5× the MIC crizotinib exposures compared to that in the control group (Fig. 4F and G). Therefore, crizotinib interferes with pyrimidine metabolism to decrease DNA synthesis and thus inhibits bacterial growth.

### Crizotinib directly targets PyrG to disrupt pyrimidine metabolism pathway.

**FIG 5:**
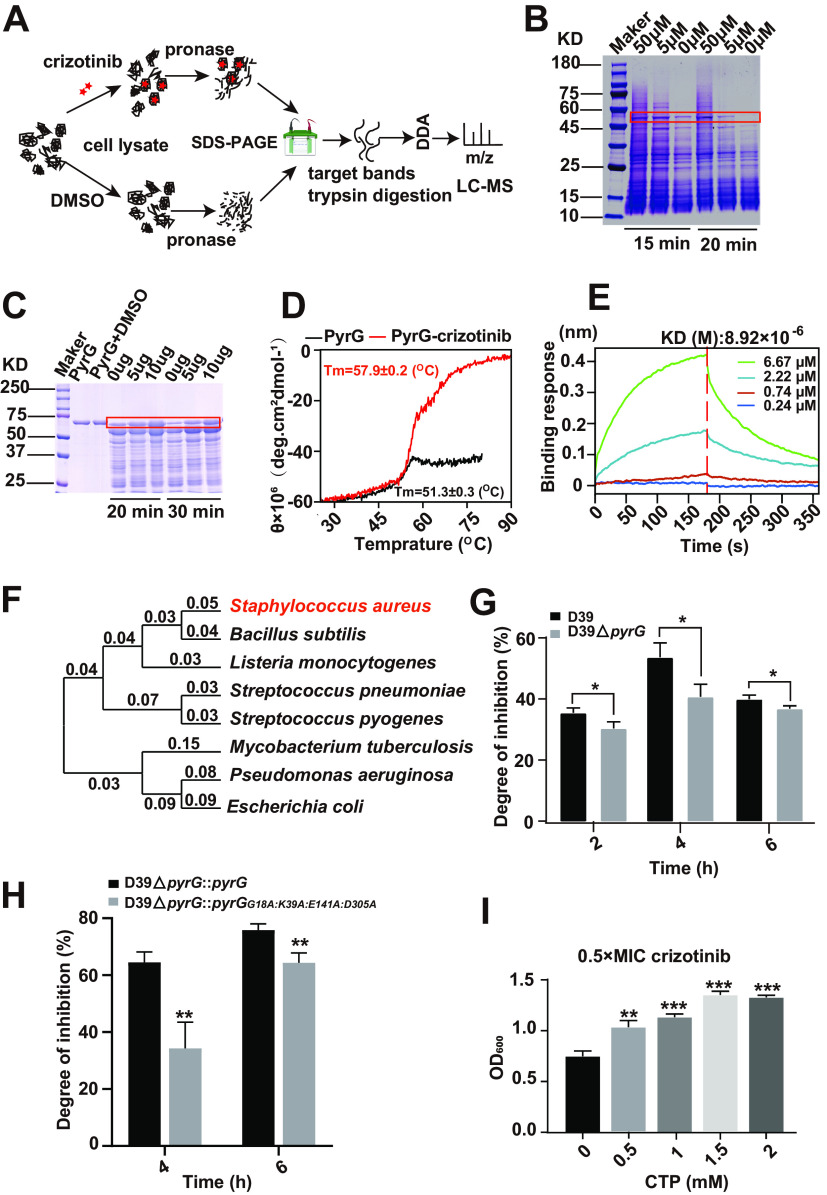
Crizotinib directly targets PyrG. (A) Schematic diagram of DARTS technology; (B) pronase digestion of the total proteins of S. aureus Newman incubated with crizotinib or DMSO; (C to E) DARTS, thermal stability, and BLI assays showing the binding between crizotinib and PyrG; (F) species evolutionary tree; (G) growth inhibition by crizotinib of the D39 WT and D39 Δ*pyrg* strains in 2, 4, and 6 h; (H) growth inhibition by crizotinib of the D39 Δ*pyrG*::*pyrG* and D39 Δ*pyrG*::*pyrG*^G18A K39A E141A D305A^ strains in 4 and 6 h; (I) OD_600_ determined after supplementation with a series of CTP concentrations of S. aureus Newman treated with 0.5× the MIC of crizotinib. Error bars indicate SD. *, *P* < 0.05; **, *P* < 0.01; ***, *P* < 0.001.

Given the efficient antibacterial effect of crizotinib, we further identified the potential target of crizotinib in S. aureus using DARTS technology (Fig. 5A). After pronase digestions for 15 min and 20 min, a band with a size of approximately 59 kDa was increased in the crizotinib-treated cell lysates from S. aureus Newman, compared to that in untreated bacteria, in a dose-dependent manner (Fig. 5B). The protein band was identified as PyrG using mass spectrometry (MS) analysis. We further confirmed the binding of crizotinib to PyrG with DARTS, thermal stability, and biolayer interferometry (BLI). Crizotinib supplementation was shown to improve the resistance of PyrG to pronase digestion (Fig. 5C), and PyrG incubated with crizotinib showed higher thermal stability than the control (Fig. 5D). Furthermore, BLI revealed crizotinib binds to PyrG in a dose-dependent manner with an equilibrium dissociation constant (*K_D_*) of 8.92 μM (Fig. 5E). These results jointly illustrated that crizotinib can directly bind to PyrG *in vitro* with high affinity.

To further validate this drug target *in vivo*, we attempted to knock out *pyrG* in S. aureus Newman several times, but failed. However, we found that S. aureus and S. pneumoniae were closely related (Fig. 5F), and crizotinib also exhibited good antibacterial activity against S. pneumoniae D39 (Fig. 1B). Fortunately, our group has a successful gene knockout model of S. pneumoniae D39. Therefore, *pyrG* was deleted in S. pneumoniae D39, and subsequently the antibacterial effect of crizotinib on S. pneumoniae D39 growth was evaluated. The results indicated that the inhibition efficiency of crizotinib against S. pneumoniae D39 Δ*pyrG* was lower than that against S. pneumoniae D39 (Fig. 5G). What’s more, to validate the drug target sites in PyrG, *pyrG* and *pyrG*^G18A K39A E141A D305A^ were introduced into D39 Δ*pyrG*. The inhibition efficiency of crizotinib against D39 Δ*pyrG*::*pyrG*^G18A K39A E141A D305A^ was also lower than that against D39 Δ*pyrG*::*pyrG*, further confirming that the crizotinib binding site of PyrG is composed of Gly18, Lys39, Glu141, and Asp305 (Fig. 5H).

These combined data further confirmed that crizotinib can directly target the key protein of CTP synthase, PyrG, in the pyrimidine metabolism pathway. Coincidently, the above proteomics data also showed that crizotinib interferes with pyrimidine metabolism and eventually disturbs DNA synthesis. Therefore, we tried to use cytidine-5′-triphosphate disodium salt (CTP) to reverse bacterial growth. A series of CTP concentrations were added to S. aureus Newman treated with 0.5× the MIC of crizotinib. The OD_600_ values indicated that CTP efficiently promoted bacterial growth (Fig. 5I). Thus, both *in vitro* and *in vivo* data indicate that crizotinib inhibits Gram-positive bacteria by hindering pyrimidine metabolism through targeting PyrG.

**FIG 6:**
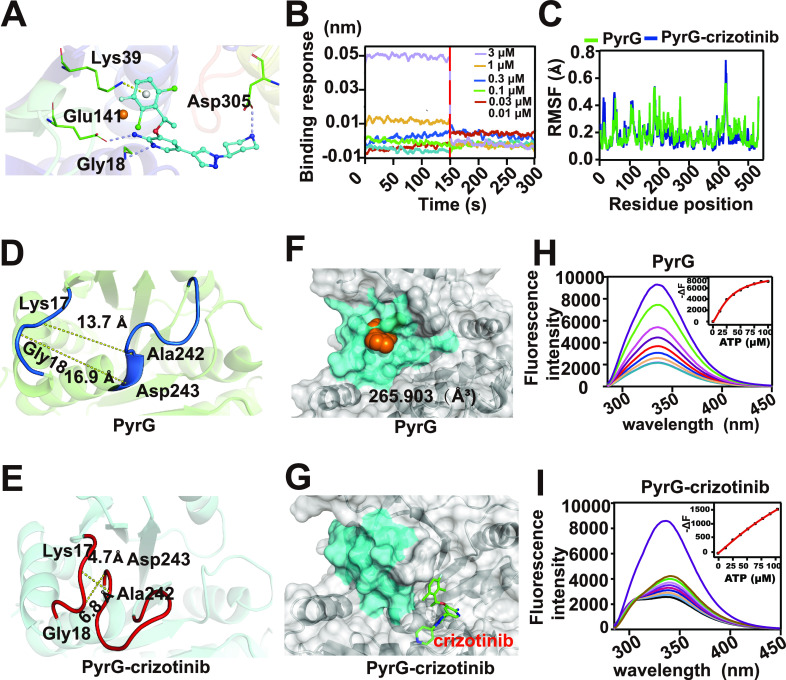
Crizotinib binding induced a conformational change of PyrG. (A) Schematic representation of the interactions between PyrG and crizotinib analyzed by UCSF Dock. Asp305, Gly18, Glu141, and Lys39 are located in the binding site. (B) BLI assay of the binding between crizotinib and PyrG^G18A K39A E141A D305A^; (C) RMSF values of all residues of PyrG-crizotinib and PyrG proteins calculated over the 100-ns trajectory; (D, E) distances between ATP-binding residues in PyrG (navy) and PyrG-crizotinib complex (red) measured by PyMOL; (F, G) closing of the ATP binding pocket in PyrG induced by crizotinib binding. The residues of the ATP binding pocket in PyrG and PyrG-crizotinib complex are colored in blue. The structures were visualized at 90 ns during molecular dynamics simulation; (H, I) fluorescence titration showing the interaction between ATP and PyrG-crizotinib complex or PyrG.

To further determine the binding mode of crizotinib and PyrG in detail, we carried out molecule docking (MD) with University of California—San Francisco (UCSF) Dock 6.9. The poses with the lowest binding free energy and the highest scoring orientation were selected for further analysis. The MD results revealed that the hydrogen atom of crizotinib directly binds to the amino group and carbonyl groups of PyrG through hydrogen bonding interactions with Gly18, Lys39, Glu141, and Asp305 of PyrG (Fig. 6A). To further verify the importance of these four residues in the binding of PyrG with crizotinib, we mutated all these residues to alanine simultaneously to eliminate the hydrogen bond between PyrG and crizotinib. A subsequent BLI assay suggested that the mutant protein PyrG^G18A K39A E141A D305A^ did not interact with crizotinib (Fig. 6B). Taken together, our data verified that Gly18, Lys39, Glu141, and Asp305 are important for PyrG binding to crizotinib.

To investigate the change in PyrG conformation upon crizotinib binding, we performed molecular dynamics simulation (MDS) with the initial conformation of the PyrG and PyrG-crizotinib postdock complex for 100 ns. Generally, most residues of PyrG showed higher root mean square fluctuation (RMSF) values than those of the PyrG-crizotinib complex (Fig. 6C), indicating that crizotinib binding results in a compact structure of PyrG. Interestingly, the amino acid distance on both sides of the ATP binding pocket in the PyrG-crizotinib complex was significantly smaller than that in PyrG, including for Ala242 and Lys17 or Asp243 and Gly18 (Fig. 6D and E). In the PyrG structure, there is a smaller solvent-accessible surface to bind ATP. We further visualized the hydrophobic surface by PyMOL and found that the ATP binding pocket was almost completely closed in the PyrG-crizotinib complex, while it remained accessible in PyrG, with a volume of 265.903 Å^3^ (Fig. 6F and G). This result suggests that crizotinib binding may lead to decreased ATP binding ability of PyrG. Subsequent fluorescence titration showed that ATP titration caused a significant fluorescence quenching of PyrG at around 335 nm, with a *K_D_* of 320 μM. Contrastingly, hardly any change in the fluorescence intensity of the PyrG-crizotinib complex was observed (Fig. 6H and I). This result was consistent with the observation from MD analysis that indicated that crizotinib binding resulted in the disappearance of the ATP binding pocket. Because ATP is the necessary substrate for PyrG to produce CTP in pyrimidine metabolism, these results may indicate that crizotinib binding to PyrG may lead to the inability to function in pyrimidine metabolism, resulting in the reduction of CTP production.

## DISCUSSION

Bacterial infection is an intractable complication in patients (19–21). S. aureus strains, especially MRSA, are often the main pathogen of clinical infection and have high incidence and mortality worldwide (22, 23). Abuse of antibiotics has led to a rapid increase in the number and type of drug-resistant bacteria, especially the emergence of superbugs, which has made it critical for the development of novel and more effective strategies to treat bacterial infections (24, 25).

Drug repurposing has been a research hot spot in the field of antibacterial drug discovery, providing a faster and more effective approach to identify new antimicrobial agents. Here, we found that the repurposed drug crizotinib, an FDA-certified novel oral multitargeted tyrosine kinase inhibitor, could inhibit Gram-positive bacteria with a broad spectrum of antibacterial activity, but scarcely had antibacterial activity against Gram-negative bacteria, which might be a result of the differences in the composition and structure of the cell wall. The unique structure and component of outer membrane in Gram-negative bacteria have selective permeability to molecules, thus reducing the sensitivity of bacteria to certain drugs. Our study provides evidence that crizotinib not only exhibits a strong antibacterial effect against sensitive Gram-positive and single-antibiotic-resistant bacteria, but also has a strong effect against clinically isolated multidrug-resistant bacteria. In addition, the animal model results showed that after treatment with crizotinib, the survival rate of mice with pneumonia increased significantly, and the pulmonary inflammation of the crizotinib-treated group was obviously reduced compared with that of the vehicle-treated mice. These results suggest that crizotinib has a high antibacterial efficacy *in vitro* and *in vivo*. Because of its antimicrobial and anticancer effects, it may be suitable for the treatment of lung cancer patients with infections caused by Gram-positive bacteria.

Crizotinib shows significant antimicrobial effects against clinical isolate strains with multiple-antibiotic-resistance phenotypes, implying that the action mechanism of crizotinib is unique compared to that of traditional antibiotics. DIA-based quantitative proteomics and biochemical verification revealed that crizotinib treatment leads to a reduction in ATP production by hindering the conversion of pyruvate to acetyl-CoA, limiting the availability of acetyl-CoA for the TCA cycle. Crizotinib treatment also disturbs pyrimidine metabolism and eventually reduces DNA synthesis. Meanwhile, in DARTS, BLI, and thermal stability assays, we found and validated the target protein of crizotinib, PyrG, which is a key CTP synthase. PyrG uses ATP as the substrate and is involved in pyrimidine metabolism, which is an important pathway for DNA synthesis (19). This antibacterial mechanism is unique compared to available antibiotics, which may explain why S. aureus exhibited low resistance to crizotinib during the process of serial passages. Moreover, a previous study has also reported that PyrG is a drug target of thiophene carboxamide derivatives to inhibit the Mycobacterium tuberculosis (20). These results suggest that PyrG may be a target for antibacterial drugs to inhibit bacteria.

Furthermore, MD and site-direct mutagenesis suggest that Gly18, Lys39, Glu141, and Asp305 are important residues for binding to crizotinib *via* hydrogen bond formation. MDS analysis showed that crizotinib binding to PyrG results in closing of the ATP binding pocket of PyrG, which causes loss of ATP binding ability and function in CTP production of PyrG. Understanding the mechanism is beneficial for the design of antibacterial drugs.

**FIG 7:**
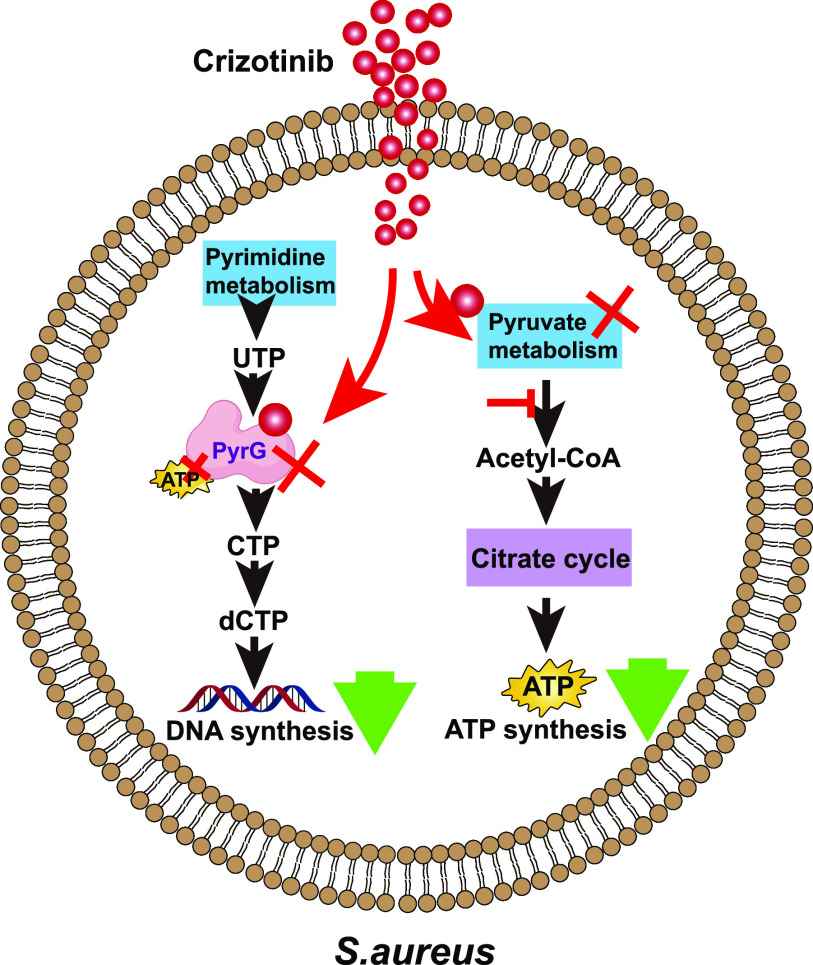
Diagram showing that crizotinib inhibits S. aureus by reducing ATP production and targeting PyrG in pyrimidine metabolism and then decreasing DNA synthesis.

In conclusion, we found that the repurposed drug crizotinib showed a broad-spectrum antibacterial effect against Gram-positive bacteria, even for clinically isolated multidrug-resistant bacteria. Crizotinib suppresses bacterial growth by disrupting CTP production in pyrimidine metabolism via targeting the CTP synthase PyrG, thus resulting in the closure of the ATP binding pocket of PyrG, causing loss of ATP binding ability and therefore loss of function (Fig. 7). Our study provides a preclinical rationale for the development of crizotinib for the treatment of Gram-positive infections.

## MATERIALS AND METHODS

### Bacterial strains and growth conditions.

The FDA-approved drug library and crizotinib were purchased from TargetMol (Shanghai, China) and dissolved in DMSO to a concentration of 5 mg/mL as a stock solution stored at −20°C. The antibiotics were purchased from Sigma (United States). The clinically isolated strains were collected from patients admitted to the affiliated Nanfang Hospital of Southern Medical University (Guangzhou). The bacterial strains, including P. aeruginosa ATCC 9027 and S. aureus ATCC 29213, were purchased from the American Type Culture Collection. S. aureus Newman, multiple-drug-resistant S. aureus 43300 (MRSA-43300), S. suis BM407, L. monocytogenes 19117, S. pneumoniae D39, and S. pyogenes MGAS5005 were stored at our laboratory. Cip^r^, Met^r^, Cli^r^, and Gent^r^ strains were domesticated by our laboratory. S. aureus, P. aeruginosa ATCC 9027, and S. suis BM407 strains were grown in tryptic soy broth (TSB) medium (Huankai, China), L. monocytogenes 19117 was cultured in brain heart infusion broth (Hopebio, China) at 37°C at 200 rpm. S. pneumoniae D39 and S. pyogenes MGAS5005 were cultured in Todd-Hewitt broth medium (Oxoid, United Kingdom) with 0.5% yeast extract (Oxoid, United Kingdom) at 37°C in 5% CO_2_ statically. Escherichia coli BW25113 and P. aeruginosa ATCC 9027 were grown in Luria-Bertani broth medium (formulation per liter: 10 g tryptone [Oxoid, United Kingdom], 5 g yeast extract [Oxoid, United Kingdom], 10 g sodium chloride [Guangzhou chemicalreagent, China]) at 37°C at 200 rpm. All bacterial strains were stored as a 15% glycerol stock at −80°C until used.

### Screening of an FDA-approved small-molecule library and determination of the MIC.

To obtain novel antibacterial agents, a drug library of 288 FDA-approved medications was tested for antibacterial activity. S. aureus Newman was examined with the 288 compounds individually at an identical concentration of 30 μM for 12 h in a 48-well microplate (1 mL per well), and the inhibitory effects of small molecules on cells were evaluated by an OD_600_ of <0.1.

To confirm the antibacterial activity of crizotinib, overnight cultures of all tested bacteria were diluted 1:100 and allowed to grow to the exponential growth phase, and then 3.2 × 10^6^ CFU of bacteria were incubated with a series of concentration gradients of crizotinib in a 48-well microplate (1 mL per well) at 37°C for 12 h at 200 rpm or as a stationary culture in a 5% CO_2_ incubator (MCO-170AICUVHL-PC; Panasonic, Japan). The MIC was defined as the minimal concentration of crizotinib that results in no visible bacterial growth (optical density at 600 nm [OD_600_] of <0.1). In order to investigate the effect of triphosphate disodium salt on bacterial growth, we assessed the growth states of S. aureus treated with 0.5× the MIC (5 μg/mL) of crizotinib with 0, 0.5, 1, 1.5, and 2 mM cytidine-5′-triphosphate disodium salt via microdilution in a 48-well plate, and then the OD_600_ of S. aureus was determined after 12 h. All experiments were performed in three biological replicates.

The assay of resistance development was performed as in previous research (26). S. aureus Newman was cultured in TSB with 0.5× the MIC of crizotinib or Amp for about 12 h, and the MIC value was remeasured, which was repeated for 20 passages. Amp treatment was used as positive control. The development of drug resistance was assessed by calculating the ratio of MIC*_n_* to MIC_0._ All experiments were performed in three biological replicates.

### Scanning electron microscopy analysis.

The bacterial morphology was observed via a scanning electron microscope (Philips XL-30ESEM; Philips Netherlands). S. aureus was allowed to grow to the exponential growth phase and then diluted 1:100 with TSB without or with crizotinib at final concentrations of 1× the MIC and incubated at 37°C. After 4 h of culture, bacteria were collected by centrifugation, washed with PBS three times, then fixed with 2.5% glutaraldehyde for 4 h and dehydrated with ascending concentrations of ethanol (30%, 50%, 70%, 80%, 90%, and 100%) for 10 min, and finally freeze-dried and coated with gold. The ultrastructure was observed by SEM.

### Determination of the synergistic effect of crizotinib with known antibiotics.

To achieve better antibacterial effects of crizotinib, the antibiotics commonly used in clinical treatment, such as gentamicin (Gent), erythromycin (Erm), clindamycin (Cli), and ampicillin (Amp), were combined with crizotinib to treat bacteria. The MICs of antibiotics against S. aureus Newman were tested the same way as for crizotinib. A checkerboard assay was performed in 48-well microplates by a method previously described (27). S. aureus Newman at the exponential growth phase was diluted 1:100 with TSB containing crizotinib (0.0625× the MIC, 0.125× the MIC, 0.25× the MIC, and 0.5× the MIC) combined with Gent, Erm, Cli, and Amp (0.0625× the MIC, 0.125× the MIC, 0.25× the MIC, and 0.5 × the MIC) and further cultured for 12 h at 37°C. The fractional inhibitory concentration index (FIC) was calculated by the formula: FIC = [MIC_A_ (combination)/MIC_A_ (alone)] + [MIC_B_ (combination)/MIC_B_ (alone)] and interpreted as follows: synergistic is an FIC of ≤0.5, additive is an FIC of >0.5 and ≤1.0, indifferent is an FIC of >1.0 and <2.0, and antagonistic is an FIC of >2.0 (28). All experiments were performed in three biological replicates.

### Mouse infection model for investigation of drug efficiency.

BALB/c female mice (6 to 8 weeks old) were purchased from the Department of Experimental Animal (Beijing HFK Bioscience) and cared for under standard conditions according to institutional guidelines. All animal experiments were approved by the Ethics Committee for Animal Experiments of Jinan University.

The mouse pneumonia model infected with the clinically isolated strain MRSA-166138 was created as reported previously (29). In brief, mice were anesthetized with 2,2,2-tribromoethanol and 2-methyl-2-butanol and then inoculated intranasally with 50 μL bacterial suspension and kept upright for 60 s to allow the bacteria to be inhaled into the lungs. The PBS was used as a negative control. The mice were treated with 15 mg/kg crizotinib or vehicle by gavage twice a day after MRSA-166138 infection for 12 h.

For the detection of survival rate, the mice (6 mice each group) were challenged with about 1 × 10^10^ CFU of the MRSA-166138 strain, and the survival status was examined at 12-h intervals for 5 days. For the investigation of lung bacterial loading and histopathology, the mice (10 mice each group) were challenged with 1 × 10^8^ CFU of MRSA-166138 for treatment for 48 h. We assessed the pulmonary inflammation distribution in the whole lung through computed tomography (CT) scan (Pingseng Healthcare, China). To determine the lung bacterial load, mice were euthanized by cervical dislocation. The lungs were homogenized, and the bacteria were counted as in previous research (29). Briefly, the whole lung of mouse was ground with 1 mL PBS, and the grinding liquid was serially diluted and spread on TSB agar plates. All plates were cultured at 37°C, and bacterial colonies were counted after 14 h of culture. For the histopathology analysis, the lungs of the mice were fixed with 4% formalin, stained with hematoxylin-eosin (HE), and then analyzed by light microscopy. In order to evaluate drug safety, the livers and kidneys were also collected for histological analysis. The level of ALT in the mouse serum was determined according to the protocol provided by the manufacturer (HuiLi Biotech, Ltd., Changchun, China).

### Drug affinity responsive target stability.

Drug affinity responsive target stability (DARTS) was performed to find out the drug target as described previously (30). Cell lysates (150 μg) from S. aureus Newman collected in the exponential phase were mixed with crizotinib or DMSO for 3 h and then incubated with 4 μg pronase (Roche, Switzerland) for 15 or 20 min at room temperature. After pronase digestion, the samples were separated by 4 to 12% SDS-PAGE gels, and then the gel was stained with Coomassie blue. The potential target protein bands were cut out from 4 to 12% SDS-PAGE gels and digested with trypsin for the mass spectrometry detection. DARTS was also performed using purified PyrG protein to verify the potential target for crizotinib.

### Cloning, expression, and purification of the PyrG and PyrG^G18A K39A E141A D305A^ protein.

The *pyrG* gene was amplified from S. aureus Newman genomic DNA. (The primers are listed in Table 2.) The pET-28a-*pyrG* plasmid was constructed as described previously (31) and verified by sequencing (Tsingke Biotech, China), and then it was transferred into E. coli BL21(DE3). When the OD_600_ of BL21/pET-28a-*pyrG* reached 0.6 to 0.8, 0.5 mM IPTG (isopropyl-β-d-1-thiogalactopyranoside) (Sigma, USA) was added, and the mixture was further cultured for 5 h at 37°C. The harvested cells were suspended in binding buffer (100 mM Na_3_PO_4_·12 H_2_O, 100 mM NaCl [pH 7.4]) and lysed by sonication on ice. The fusion protein was isolated by Ni-nitrilotriacetic acid (NTA) agarose (Protino, Germany). The mutant PyrG^G18A K39A E141A D305A^ protein was constructed by site-directed mutagenesis according to the manufacturer’s instructions (Vazyme, China) (32).

**TABLE 2:** Primers used in this study

Application and primer name	Sequence (5′→3′)
Protein expression	
pyrG-F-Newman	CGGGATCCATGACAAAATTTATTTTTGTAACAG
pyrG-R-Newman	GGGGTACCTTTATTTTGTTGATATTTTAATGAAG

Mutant protein	
39-F-Newman	ATTCAAGCATTCGATCCATACTTAAATGTTGACCC
39-R-Newman	GGATCGAATGCTTGAATTGTTACATTTAGACCTCTATCTTT
141-F-Newman	TATCACTGCAATTGGCGGTACAACAGGTGATAT
141-R-Newman	CGCCAATTGCAGTGATAACAACGTCTGCATTCGT
18-F-Newman	AGGGAAGGCTATTACAGCATCTTCTCTAGGTAGATTATTAAA
18-R-Newman	CTGTAATAGCCTTCCCTAATGATGAAACTACGCC
305-F-Newman	GTTAGCTTACAAGCTGCATATTTATCAGTTGTTGAATCATTGA
305-R-Newman	GCAGCTTGTAAGCTAACATATTTACCTACTAAACCA

Mutant strain	
18-F-D39	TGGGAAAGCAATTGTGGCAGCGAGTCTAGG
18-R-D39	CCACAATTGCTTTCCCAATAGACGATACCACA
39-F-D39	CATTCAAGCATTTGACCCTTATATCAATATTGATCCGGGAA
39-R-D39	GGTCAAATGCTTGAATGGTTACTTTGAGACCACGATTTTTCA
141-F-D39	TATCACAGCAGTTGGTGGAACAGTAGGAGATATCG
141-R-D39	CACCAACTGCTGTGATAATGACATCAGAGTCGGTCGTTAG
305-F-D39	AGTTGCAAGCAGCCTATATCTCAGTGGTCGAAGCC
305-R-D39	ATATAGGCTGCTTGCAACTCCACATACTTACCAACAAGG
pyrG-F-D39	CGGAATTCATGTCTACGAAATATATTTTTGTAACTGGTGGTGT
pyrG-R-D39	CGGGATCCCTAATTGCTGTTCTCAACCGCTGC

Gene knockout	
erm-F	CCGGGCCCAAAATTTGTTTGAT
erm-R	AGTCGGCAGCGACTCATAGAAT
pyrG-up-F-D39	TAGTATAAATGAGGAGAAACGCTTTGGAATTAGAAGT
pyrG-up-R-D39	ATCAAACAAATTTTGGGCCCGGAGATTCCTCTTTCTAAAATGCTCAAGGTCT
pyrG-down-F-D39	ATTCTATGAGTCGCTGCCGACTCAAAATCAGAACCTTTGAGAAAAATCTCAGAGG
pyrG-down-R-D39	CTCTCAGCCAAAAAGAAAGTGAAGTGC

RT-qPCR	
pyrC-F	GTGGTGCAATGCATGAAGGG
pyrC-R	CATGAATGCCTGCGCGTTTA
pyrB-F	TCACGTGTCGCACGTAGTAA
pyrB-R	TTCTGCAAGCCCATGCCTTT
pyrF-F	GCTGCTGGTGGCGTAAAAAT
pyrF-R	GAGGTGAACAAACAACGCCA
pyrE-F	TGCAACAGCTGGTATTCCACA
pyrE-R	AGGCTTCAACTGCTGTGACT
pyrD-F	ACACCTTGGATTCGGTGCTT
pyrD-R	ACGCGCTTCATAAGGTGTCA
pyrAA-F	TAGCAATGGCTCCAGATGGC
pyrAA-R	TTCGCACCACGATGACCAAA

### Thermal stability assay.

Thermal stability of protein was studied by on circular dichroism spectrometer (Chirascan, United Kingdom) as described previously (33). The protein samples with or without 10 μM crizotinib were prepared at a concentration of 3 μM in PBS (pH 7.2). The data were recorded at 223 nm for PyrG and PyrG^G18A K39A E141A D305A^ with continuous heating at 2°C/min. At the endpoint, the temperature transition was immediately reversed to cool the sample down at a constant rate of 5°C/min.

### Interactions between PyrGs and crizotinib analyzed by biolayer interferometry.

The interactions between PyrGs and crizotinib were detected with biolayer interferometry (BLI) (ForteBio Octet K2). PyrG was incubated with biotin (Genemore, China) at 1:1 molar ratio, desalted by a desalting column (G-MM-IGT; Genemore, China), and then diluted in black 96-well plates (Thermo, Germany) using PBS buffer with 0.5% DMSO. The tips were prehydrated in 210 μL sample diluent for at least 10 min, and then biotinylated PyrG was loaded on super streptavidin (SSA) high-binding biosensors (Fortebio, USA). Next, SSA high-binding biosensors were incubated with an increasing concentration of crizotinib (0.24 μM to 6.67 μM) in PBS with 0.5% DMSO to generate binding and dissociation curves. The association and dissociation between crizotinib and PyrG were assessed by the shift in wavelength (nanometers) after subtracting blank responses. Raw data were obtained with Octet software (version 11.0; Menio Park, Germany).

### Evolutionary analysis.

The rRNA sequence of 16S from S. aureus Newman was used to query the NCBI database using the nucleotide BLAST tool. The multiple-sequence alignment and cluster analysis of the high-scoring rRNA were performed with the software package Clustal-X 2.1. MEGA5.05 was applied to construct the evolutionary tree.

### Construction of D39Δ*pyrG*, D39 Δ*pyrG*::*pyrG* and D39 Δ*pyrG*::*pyrG*^G18A K39A E141A D305A^ strains.

To construct the gene knockout strain D39 Δ*pyrG*, the *pyrG* gene was replaced with the erythromycin (*erm*) gene in wild-type (WT) D39 by homologous recombination. Long flanking homologous (LFH) segments containing *pyrG*-up, the *erm* gene, and *pyrG*-down were obtained by PCR and inserted into WT D39. Next, a Columbia blood agar plate with 0.25 mg/mL Erm was used to screen the positive clones. To construct a D39 Δ*pyrG*::*pyrG* strain and D39 Δ*pyrG*::*pyrG*^G18A K39A E141A D305A^ complement strain, the p169+*pyrG* or p169+*pyrG*^G18A K39A E141A D305A^ recombinant plasmid was introduced into the D39 Δ*pyrG* strain. The positive clones were screened with a 4 mg/L chloramphenicol-resistant blood agar plate. The detailed procedures of gene knockout and complementation were the same as that described in our previous studies (34). The primers are listed in Table 1. Then, overnight cultures of the WT D39, D39 Δ*pyrG*, D39 Δ*pyrG*::*pyrG*, and D39 Δ*pyrG*::*pyrG*^G18A K39A E141A D305A^ strains were diluted 1% with THYE (Todd-Hewitt broth with yeast extract) at 37°C in a 5% CO_2_ incubator, allowed to grow to an OD_600_ of ≈0.6, and then incubated with 0.5× the MIC of crizotinib in a 48-well microplate (1 mL per well). The growth was detected at OD_600_ by UV-visible spectroscopy (Thermo, USA).

### Measurement of growth curves of S. aureus.

The growth curves of S. aureus Newman cells untreated or treated with crizotinib were detected as in previous research (35). S. aureus Newman cells were incubated with various concentrations of crizotinib (corresponding to 0× the MIC, 0.5× the MIC, and 1× the MIC) in a 48-well microplate (1 mL per well) for 12 h, and OD_600_ was determined by microplate reader (BioTek Epoch, USA). The data were analyzed by Prism 6.0 (GraphPad Software, USA). All experiments were performed in three biological replicates.

### Protein preparation and DIA quantitative proteomics analysis.

When S. aureus Newman grew at an OD_600_ of ≈0.2, 0.5× the MIC of crizotinib was added to the medium, and the mixture was further cultured for 1 h or 2 h, which were defined as the 1-h group and 2-h group, respectively. The untreated bacteria were collected at the same time points.

Protein extraction, digestion, and desalting were performed as described previously (35). All experiments were performed in three biological replicates. Data-dependent acquisition and DIA MS analyses were performed on an Orbitrap Fusion Lumos mass spectrometer (Thermo, USA). Raw data were analyzed with Proteome Discoverer 2.4 (Thermo, USA) and Spectronaut 11 (Biognosys AG, Switzerland). The search parameters were set as follows: MS tolerance, 10 ppm; number of trypsins with missed cleavage, 2; fragment mass tolerance, 0.02 Da; enzyme, trypsin; carbamidomethylation (Cys, +57.021 Da) of cysteine as a fixed modification; and oxidation of methionine as a variable modification. The proteins were considered for further analysis following three criteria: protein-level false-discovery rate (FDR) of ≤1%, number of unique peptides of ≥1, and peptide length of ≥7 amino acids (aa). DEPs were considered as follows: a *P* value of <0.05 and |fold change| of ≥1.5. Kyoto Encyclopedia of Genes and Genomes (KEGG) pathway enrichment analysis was performed on the DEPs to identify overrepresented biological pathways using the ClueGO (version 2.3.3) program and visualized with Cytoscape (version 3.7.1). The *P* value of the pathway enrichment was calculated based on the right-hypergeometric test and corrected with Bonferroni’s adjustment. Herein, the *P* value was set as two-sided hypergeometric tests and adjusted using Bonferroni’s correction, and a *P* value of <0.05 was considered statistically significant (36).

### Quantification of ATP and acetyl-CoA levels.

S. aureus Newman cells were cultured, harvested, and lysed by the method described above in the proteomics study. The content of ATP was quantified using an enhanced ATP assay kit (Beyotime, China) in accordance with the manufacturer’s instruction. The supernatant was mixed with detection solution quickly, and the luminescence was detected using a GloMax luminometer (Promega, USA). The measurement of the acetyl-CoA level was carried out according to the protocols of the acetyl-CoA content assay kit (Solarbio, Beijing, China). The absorbance at 340 nm of the supernatant mixed with detecting solution was determined by UV spectrophotometer (Evolution 300; Thermo, United Kingdom). All experiments were performed in three biological replicates.

### RT-qPCR.

Total RNA was extracted from S. aureus Newman treated with 0.5× the MIC of crizotinib for 1 and 2 h using the Eastep super RNA extraction kit (Promega, USA) according to the manufacturer’s protocol. The mRNA was reverse transcribed using a TransScript II one-step RT-PCR SuperMix kit (TransGen, China). Real-time quantitative PCR (RT-qPCR) was performed with the TransStart Tip Green qPCR SuperMix kit (TransGen, China). The expression of target genes was assayed using the StepOne system (Applied Biosystems). The fold changes of selected genes were normalized to the threshold cycle (*C_T_*) value of 16S RNA amplified from the corresponding sample and calculated using the 2^−ΔΔ^*^CT^* method (37). The primer sequences are listed in Table 1. All experiments were performed in three biological replicates.

### DNA detection.

The general DNA indicator DAPI (C1002; Beyotime, China) was utilized to examine the DNA content in S. aureus Newman cells untreated or treated with crizotinib for 2 h. Briefly, the collected S. aureus cells were incubated with DAPI (10 μg/mL) for 30 min at 37°C in the dark and washed with prechilled PBS three times. The DNA content in S. aureus cells was detected with a flow cytometer (BD FACS Celesta; Becton, Dickinson, USA). DAPI was excited by a 364-nm laser, and fluorescence emission at 454 nm per 10,000 cells was analyzed and processed with FlowJo (version 10.0). Mean fluorescent intensity (MFI) was used to determine fold change between the crizotinib treatment group and control group. All experiments were performed in three biological replicates.

### Homology modeling and molecule docking.

For homology modeling, the NCBI BLAST search tool was used to find several proteins with the highest homology to PyrG (PDB no. 4ZDK). Then, SWISS-MODEL (https://Swissmodel.expasy.org/) was used to model the protein structure of PyrG. Energy minimization was carried out to optimize the structure by using Chimera, and quality assessment was performed by MolProbity (Molprobity.biochem.duke.edu). For molecule docking, Dock 6.9 (https://dock.compbio.ucsf.edu/) and Chimera (https://www.cgl.ucsf.edu/chimera) of UCSF were used to generate the grid box with the built model. In addition, the pose with the best score was selected to carry out the PyrG-crizotinib interaction analysis. Finally, the Protein-Ligand Interaction Profiler and PyMol (https://pymol.org/edu/) were used to perform the PyrG-crizotinib interaction analysis and draw the image of the docked pose.

### MDS and trajectory analysis.

Molecular dynamics simulation (MDS) was carried out in the 100-ns trajectory by the Gromacs 2021.4 (https://www.gromacs.org) package with the SPC water model in the Gromos43a1 force field. The neutralizing ions (Na^+^) and chloride (Cl^−^) were added to neutralize the systems. Then, the homology modeling structures of PyrG, PyrG^G18A K39A E141A D305A^, and PyrG-crizotinib complex were used as starting conformations in one cubic periodic box, and the minimum distance between the protein and the box boundary was set at 1.0 nm. The MDS cascade of minimization, heating, equilibration, and production was subsequently executed. A 100-ns dynamic calculation was performed at 0.1 MP and 298 K in this system. The RMSF of each residue was calculated using the gmx rmsf tool, and the short-range interaction energy was calculated with the GROMACS energy module. The last 50-ns trajectory of PyrG, PyrG^G18A K39A E141A D305A^, and PyrG-crizotinib complex was extracted to visualize the conformation change during MDS. GraphPad Prism 8.0.2 software were used to draw the structure diagram (34).

### Fluorescence titration.

Fluorescence titration was performed in fluorescence spectra (Hitachi F7000, Japan). Two microliters of 10 mM ATP was gradually added to 1.5 mL PyrG protein solution (10 μM), and the mixture was incubated for 1 min at 37°C before each measurement. The excitation wavelength was 275 nm, and the emission wavelength was from 285 nm to 450 nm. The fluorescence intensity at 335 nm was extracted to produce the titration curve. The dissociation constants (*K*_D_) of proteins with ATP were calculated according to the method reported in the previous studies (38, 39).

### Statistics.

Data were analyzed by two-tailed, unpaired Student *t* tests and are expressed as means and standard deviations (SD). Statistical analysis was conducted using Prism 6.0 (GraphPad Software, USA). Results were considered significant at *P* values of <0.05.

### Data availability.

The raw proteomic data and search results have been deposited to the ProteomeXchange Consortium via the PRIDE (40) partner repository and can be accessed with the reviewer account at http://www.ebi.ac.uk/pride under accession no. PXD029546.
